# Novel Approach for Endoscopic Management of Duodenal Injury during Perirenal Infected Haematoma Drainage after Shock-Wave Lithotripsy

**DOI:** 10.1155/2018/2020572

**Published:** 2018-06-13

**Authors:** Nariman Gadzhiev, Dmitry Gorelov, Alexander Smirnov, Salman Al-Shukri, Sergei Petrov

**Affiliations:** ^1^Department of Urology, Pavlov First Saint Petersburg State Medical University, Saint-Petersburg, Russia; ^2^Department of Endoscopy, Pavlov First Saint Petersburg State Medical University, Saint-Petersburg, Russia; ^3^Department of Urology, The Nikiforov Russian Center of Emergency and Radiation Medicine, Saint-Petersburg, Russia

## Abstract

*Background. *Gaining percutaneous access during percutaneous nephrolithotomy (PNL) can be complicated with the bowel injury. We report a novel approach of management of duodenal injury complicating percutaneous drainage of infected haematoma after Shock-Wave Lithotripsy (SWL).* Case Presentation. *A 57-year-old patient with the 15 mm right pelvic kidney stone underwent uneventful SWL. Patient visited emergency department 3 days later with high fever and chills with severe right flank pain. CT urography revealed lower pole kidney injury with signs of infected hematoma due to low attenuation areas but without signs of obstruction or urine leakage. Infected haematoma was drained percutaneously under ultrasound and X-ray control and a pigtail catheter 10 Fr was left beneath the lower pole of the right kidney. Postoperatively duodenal injury was suspected due to amber color, low creatinine, and high bilirubin level in the drainage output. CT demonstrated that the pigtail of the drain had entered the second part of the duodenum. Catheter was withdrawn and defect of the duodenal wall was stapled with four staples endoscopically. After 2 days of fasting patient was allowed to start oral food intake and was discharged on the 5th day.* Conclusion*. Injury of the duodenum during percutaneous kidney manipulation is an extremely rare complication. Conservative management consisting of endoscopic stapling of the duodenal wall defect is a safe and feasible approach to expediting the recovery of the patient.

## 1. Introduction

Shock-wave lithotripsy (SWL) is one of the treatments of choice for all kidney stones up to 2 cm and up to 1000 HU of stone density. The most common complications of SWL are minor, such as renal colic or “steinstrasse” and not exceeding 7%. Major complications are rare and include renal injury with hematoma and sepsis [1]. Infected hematoma may need percutaneous drainage [2], which in our case was complicated with duodenal injury. To our knowledge, we present the first case report of a successful endoscopic management of a duodenal injury that occurred during the percutaneous drainage of infected hematoma in the lower pole of the right kidney.

## 2. Case Report Presentation

**Figure 1:**
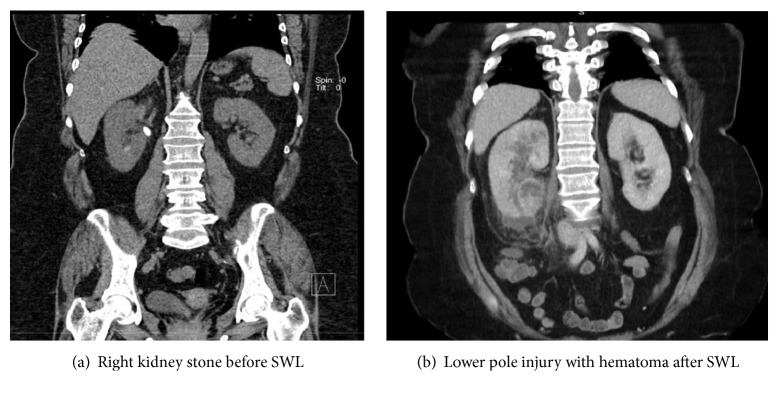


A 57-year-old patient with right flank pain was presented. A low-dose CT revealed a 15 mm stone at the right ureteropelvic junction causing hydronephrosis. Kidney stone density was 940 HU (**Figure 1(a))**. Patient received one session of SWL: 3000 pulses with a frequency of 70/min at an 85% of maximal voltage (Dornier Lithotripter-S, Germany). Routine perioperative imaging showed effective disintegration of the stone and the patient left the hospital in a good general condition. The patient was seen 3 days after SWL with complaints of right flank pain and fever with chills. A kidney ultrasound revealed a hematoma at the lower pole of the right kidney. Laboratory evaluation showed severe leukocytosis with extremely elevated acute-phase proteins. A CT urography demonstrated a lower pole kidney injury with hematoma without signs of obstruction or urine leakage (**Figure 1(b)**).

**Figure 2:**
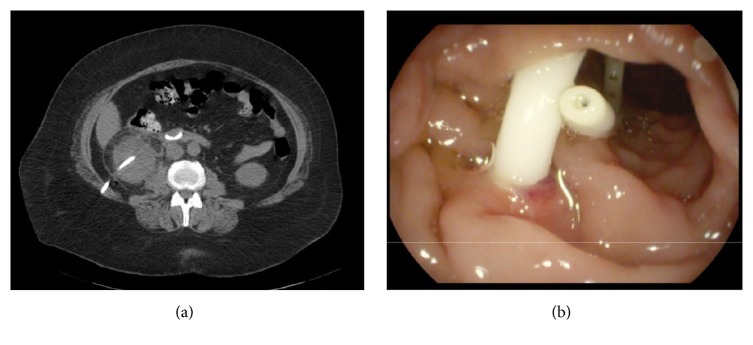
(a) CT. Percutaneous drain in the second part of the duodenum. (b). Pigtail of the percutaneous drain in the second part of the duodenum.

After obtaining urine for culture broad-spectrum antibiotics were prescribed. Despite the ongoing treatment, patient demonstrated no signs of improvement and a decision to proceed with a percutaneous drainage of the supposedly infected hematoma was made. Under local anesthesia and ultrasound control, the hematoma was drained by an experienced urologist of what appeared to be a pus-like liquid and was immediately sent for culture and pigtail catheter 10 Fr was inserted beneath the lower pole of the right kidney. Small amount of contrast injected through the catheter did not raise any concerns. During the next two hours, the drain output was about 50 ml and was amber yellow. Lab tests of the drain output showed it to be high in bilirubin and inherent to the bile. A noncontrast CT demonstrated that the pigtail of the percutaneous drain went partly through the parenchyma of the lower pole of the kidney and entered the second part of the duodenum (**Figure 2(a)**). At this point, different intervention options including open duodenal mobilization with duodenal wall defect closure were considered. After lengthy considerations, we decided to proceed with the endoscopic route. Endoscopically diagnosis was confirmed (**Figure 2(b)**).

**Figure 3:**
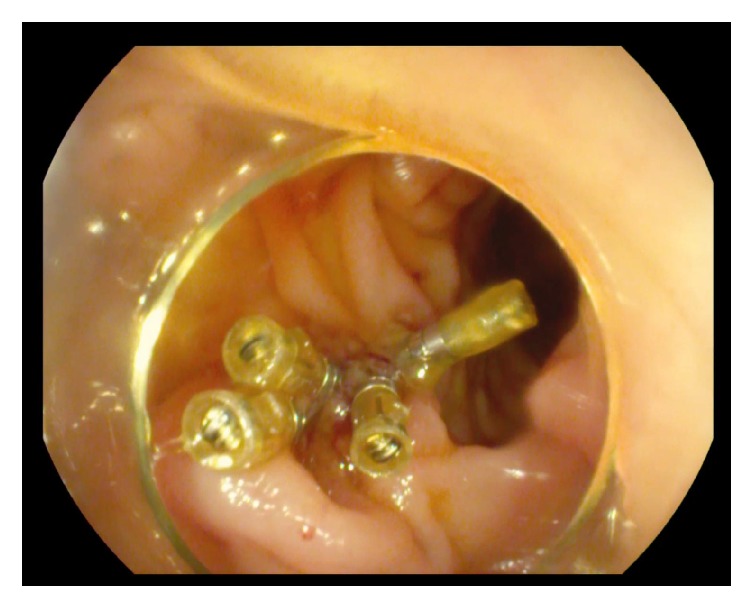
The pigtail drain has been removed and the wall defect has been stapled.

The percutaneous drain was withdrawn from the duodenum and the wall defect was stapled with four staples by the endoscopist (**Figure 3**). Considering the intraparenchymal route of the drain and the absence of the aspirate during its gradual removal, a decision was made to completely remove the drain.

**Figure 4:**
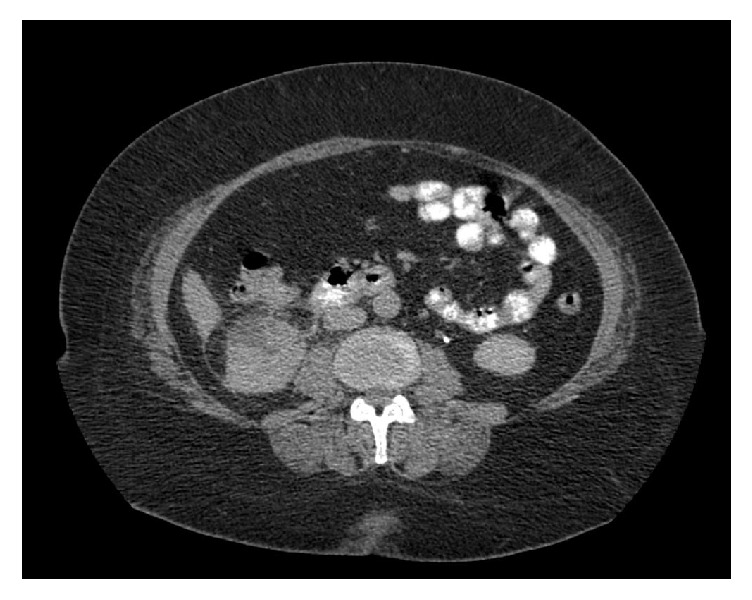
CT demonstrating no signs of contrast leakage from duodenum.

Postoperatively, the patient exhibited no peritoneal signs and was kept on* nil per os* for the following two days. Broad-spectrum antibiotics were continued, and bowel rest was achieved by the means of a nasogastric tube. The patient was allowed to start oral food intake from the third day after removal of the nasogastric tube. On the fourth day, the patient became afebrile with normalization of blood parameters. A repeat CT with oral contrast administration was performed on the 5th day where neither signs of contrast leakage at the stapling line nor stone fragments after SWL were detected.

(**Figure 4**). Thereby, the patient was successfully discharged.

## 3. Discussion

Bowel injury may occur as a complication of percutaneous nephrolithotomy (PNL) due to some technical aspects of the procedure or anatomic variations. Most often these injuries are colonic and are managed conservatively provided the injury is retroperitoneal and no signs of peritonitis are present [3]. Being intraperitoneal, the small bowel is located at a distance from the kidney, and therefore the risk of its injury during PNL is extremely low. However, the second portion of the duodenum is retroperitoneal and lies in close proximity to the lower pole of the right kidney. Usually, the duodenum is mobile and well protected in the fibrofatty tissues of the retroperitoneum [4]. However, our patient had an infected hematoma, allowing the duodenum to become adherent to the lower pole of the right kidney. Our patient received SWL session according to the existing standard protocol (power ramping, 70 pulses per minute, and 3 000 pulses for the kidney) and had no special predisposing to hematoma formation factors. In our case, a needle over advancement probably took place and this complication could be detected at the end of the manipulation by X-ray control if we injected more contrast through the catheter, but fear to exacerbate the infectious process precluded us from doing so. Management of a duodenal injury associated with percutaneous kidney access gaining was well described so far and ranged from surgical options including laparotomy to conservative, with prolonged fasting up to 14 days, parenteral hyperalimentation, and prolonged hospital stay [5, 6]. Despite the fact that the location of the duodenal defect directed our surgical team towards open surgery experience of our endoscopist, who is managing duodenal adenocarcinoma endoscopically [7] and the size of the defect was taken into consideration.

To our knowledge, this is the first clinical case demonstrating the successful endoscopic management of a duodenal injury without a periduodenal drain with fast recovery.

## 4. Conclusion

Injury of the duodenum during percutaneous kidney manipulation is an extremely rare complication. Conservative management consisting of endoscopic stapling of the duodenal wall defect is a safe and feasible approach expediting the recovery of the patient.

## Data Availability

The data used to support the findings of this study are included within the article.
